# Gender differences in a clinical sample of 60+ year old patients receiving treatment for alcohol use disorder

**DOI:** 10.1192/j.eurpsy.2022.2138

**Published:** 2022-09-01

**Authors:** J. Tryggedsson, K. Andersen, M. Bogenschutz, A. Nielsen

**Affiliations:** 1 University of Southern Denmark, Faculty of Health Sciences, Unit Of Clinical Alcohol Research, Clinical Institute, Odense C, Denmark; 2 Region of Southern Denmark, Department Of Mental Health Odense, Odense, Denmark; 3 NYU Grossman School of Medicine, Department Of Psychiatry, New York, United States of America

**Keywords:** Alcohol Treatment, Motivational enhancement therapy, Older Adults, Alcohol use disorder

## Abstract

**Introduction:**

Gender differences have been found in treatment-seeking older adults with AUD, concerning areas such as quality of life, drinking patterns, and prevalence of AUD. However, little is known about how these gender differences may relate to treatment.

**Objectives:**

To investigate gender differences in quality of life, problematic areas at treatment start, and subsequent choice of treatment, in a clinical sample of 60+ year old patients receiving treatment for AUD.

**Methods:**

We will utilize data from the Elderly Study; a multi-national (USA, Germany, and Denmark), single-blind randomized controlled trial. Participants (n=693) were randomized to brief, outpatient treatment based on motivational enhancement therapy alone (4 sessions) or motivational enhancement therapy followed by a community reinforcement approach age-adapted to older adults (up to 8 sessions). The latter was a module-based treatment where participants chose which module(s) they deemed most relevant. Modules focused on coping with aging, building sober networks, mood management, etc. The gender differences at baseline will be described by means of descriptive statistics (e.g. one-way analysis of variance, χ2 statistics, etc.). Gender differences, including choice of modules, will be investigated by means of multivariate statistics, e.g. generalized linear models. Analyses will be controlled for relevant confounders such as age, country, education, work situation, marital status, family and friends, type of housing, etc.

**Results:**

Will be presented at the EPA Congress.

**Conclusions:**

Will be presented at the EPA Congress.

**Disclosure:**

No significant relationships.

